# Mechanisms of protein toxicity in neurodegenerative diseases

**DOI:** 10.1007/s00018-018-2854-4

**Published:** 2018-06-12

**Authors:** Chang Geon Chung, Hyosang Lee, Sung Bae Lee

**Affiliations:** Department of Brain and Cognitive Sciences, DGIST, Daegu, 42988 Republic of Korea

**Keywords:** Parkinson’s disease, Alzheimer’s disease, Huntington’s disease, Polyglutamine diseases, Lou Gehrig’s disease, Amyotrophic lateral sclerosis, Frontotemporal dementia, Stress granules, Protein inclusions

## Abstract

Protein toxicity can be defined as all the pathological changes that ensue from accumulation, mis-localization, and/or multimerization of disease-specific proteins. Most neurodegenerative diseases manifest protein toxicity as one of their key pathogenic mechanisms, the details of which remain unclear. By systematically deconstructing the nature of toxic proteins, we aim to elucidate and illuminate some of the key mechanisms of protein toxicity from which therapeutic insights may be drawn. In this review, we focus specifically on protein toxicity from the point of view of various cellular compartments such as the nucleus and the mitochondria. We also discuss the cell-to-cell propagation of toxic disease proteins that complicates the mechanistic understanding of the disease progression as well as the spatiotemporal point at which to therapeutically intervene. Finally, we discuss selective neuronal vulnerability, which still remains largely enigmatic.

## Introduction

The prevalence of neurodegenerative diseases, including Alzheimer’s disease (AD), Parkinson’s disease (PD), amyotrophic lateral sclerosis (ALS), frontotemporal dementia (FTD), and Huntington’s disease (HD), is increasing at an alarming rate due to the increase in average life expectancy. Patients with these diseases display serious neurological disabilities, such as memory impairment and motor problems, for which there are no cure. One of the cardinal features of neurodegenerative diseases is the presence of protein toxicity [1]. Here, we define protein toxicity as all the pathological alterations that result from the accumulation, oligomerization, and/or multimerization of disease-associated toxic proteins.

Protein toxicity is a unifying feature of both sporadic and familial cases of neurodegenerative diseases. One of the mechanisms by which protein toxicity occurs is through genetic mutations. For example, 5 point mutations in the genes encoding *synuclein alpha* (*SNCA*; A53T, A30P, E46K, H50Q, and G51D) and 52 mutations (alzforum.org/mutations) in *amyloid precursor protein* (*APP*) have so far been identified to be associated with PD and AD, respectively [2, 3]. Likewise, protein toxicity can arise from more than 20 genetic mutations in the *TARDBP* gene encoding TDP-43 protein, which are associated with ALS and FTD [4]. In addition, a recently identified GGGGCC repeat expansion in the intronic region of the *C9ORF72* gene is associated with ALS/FTD and is known to produce five different dipeptide-repeat proteins (DPRs; poly-GA, -GR, -GP, -PR, -PA) through repeat associated non-AUG (RAN) translation [5]. The arginine-rich DPRs, in particular, have been shown to cause protein toxicity [6]. Moreover, polyQ protein toxicity is solely caused by an expansion mutation of the glutamine tract in each of the genes responsible for polyQ diseases [7].

On the other hand, aberrant proteins generated independently of known genetic mutations can also contribute to protein toxicity. For instance, abnormal cytoplasmic accumulation of TDP-43, known as “TDP-43 pathology,” is observed in most cases of ALS and in about half of FTD cases, even when there is no *TARDP* mutation [8]. Likewise, independent of *SNCA* mutation, α-synuclein aggregation is often observed in PD and several other neurological disorders known as “synucleinopathies”. In fact, PD is rarely caused by mutations in *SNCA* [9]. Similarly, AD is rarely caused by mutations in *APP * [10], yet accumulation of amyloid beta is the hallmark feature of AD. Thus, regardless of the disease etiology (sporadic or familial), protein toxicity seems to be a hallmark of most neurodegenerative diseases.

**Fig. 1:**
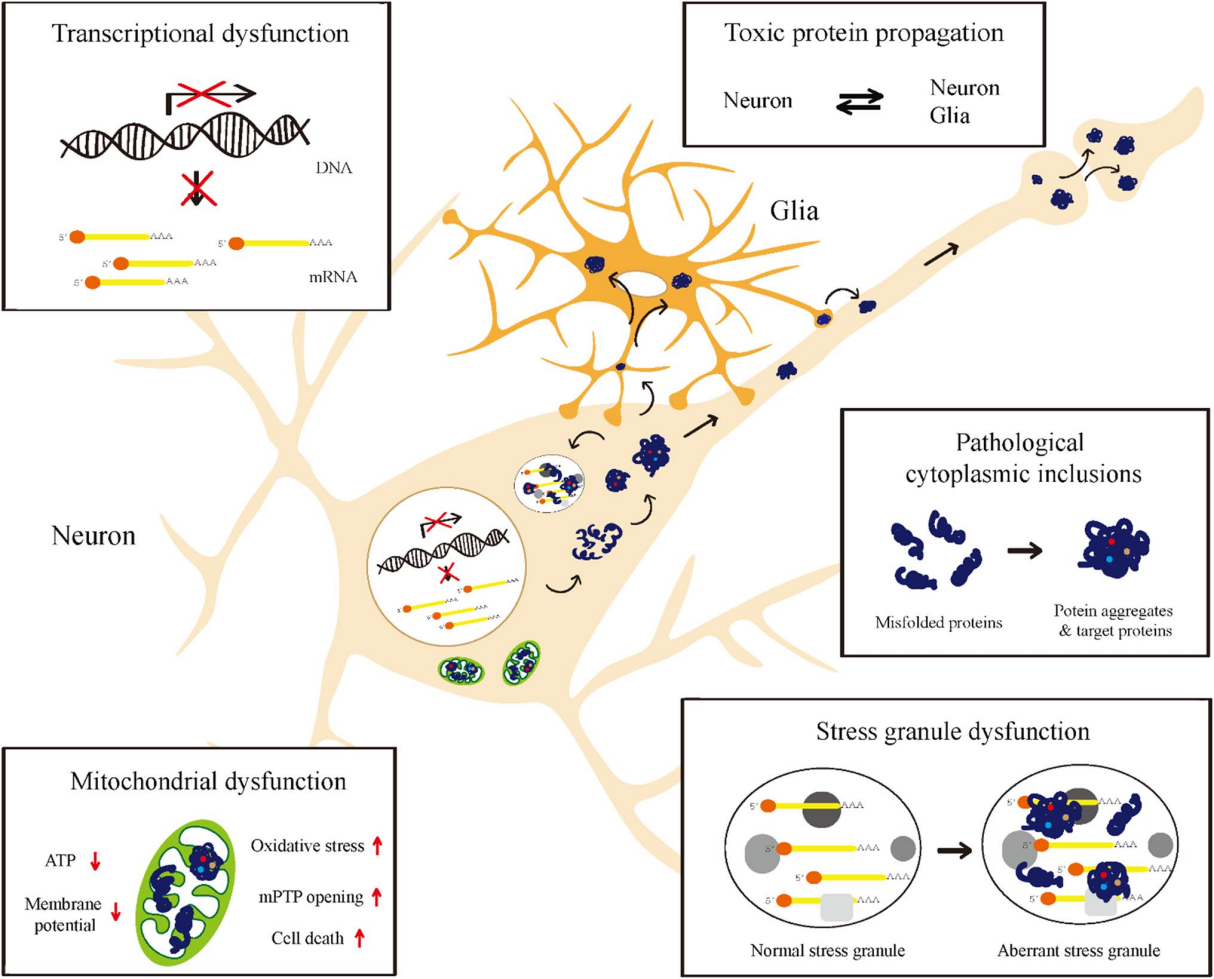
A schematic overview of protein toxicity. Accumulation of toxic disease proteins is shown to induce dysfunctions in specified compartments such as the nucleus, mitochondria, cytoplasm, and stress granules. They can also propagate into other nearby cells, spreading the disease pathology

In neurodegenerative diseases, protein toxicity in affected neurons may result in cellular defects such as transcriptional alteration, mitochondrial dysfunction, and an impaired protein/RNA quality control system, all of which critically contribute to the initiation and progression of neurodegenerative diseases. Although cell death is the final outcome of the disease process, cell death is often preceded by neurological deficits in animal models and patients [11, 12]. Hence, this review will focus on the neuronal dysfunction that occurs prior to cell death. Notably, each type of cellular defects is not absolutely specific to a certain neurodegenerative disease; but instead, these defects are more commonly observed in a variety of disease cases. Given the crucial contribution of protein toxicity to neurodegenerative disease pathogenesis, increasing our understanding of protein toxicity is indispensable for future development of rational and effective therapeutics for these diseases. Instead of characterizing protein toxicity from one disease to another (e.g., AD, PD, and ALS), in the following sections, we discuss the mechanisms underlying protein toxicity from one subcellular compartment to another (e.g., nucleus and mitochondria; see Table 1 and Fig. 1).

**Table 1:** Summary of protein toxicity based upon the subcellular localization of toxic disease proteins

Diseases	Toxic proteins	Phenotypes	Human/iPSC	Mouse	Fly	Cell culture	Others	References
**Nucleus**								
SCA3	PolyQ-expanded ataxin-3	Epigenetic and transcriptional dysfunction				O		[26, 33]
HD	PolyQ-expanded huntingtin	Epigenetic and transcriptional dysfunction, and nuclear aggregation	O	O	O	O	Sheep, rhesus monkey	[21, 22, 24, 25, 28, 30, 32, 34, 35, 41–43]
HD	PolyQ-expanded huntingtin	Nucleocytoplasmic transport dysfunction	O	O	O	O		[53, 54]
DRPLA	PolyQ-expanded atrophin-1	Mouse behavioral and survival phenotypes from histone hypoacetylation and cellular toxicity from interference of CBP- mediated transcription	O	O		O		[24, 44]
SCA1	PolyQ-expanded ataxin-1	Transcriptional dysfunction		O		O		[40]
SBMA	PolyQ-expanded androgen receptor	Cellular toxicity arising from CBP sequestration into NI	O	O		O		[26]
SCA7	PolyQ-expanded ataxin-7	CBP and RORα1-mediated transcriptional repression				O		[27]
ALS/FTD	Poly-PR repeat protein	Nucleocytoplasmic transport dysfunction			O	O	Frog *X. laevis* oocyte	[56, 57]
**Cytoplasm**								
Prion diseases	Prion protein toxic β-sheet isoforms	Blockage of substrate entry into 20S proteasome		O		O		[59]
AD	Hypophosphorylated Tau oligomers	Synaptic Tau interacts with 26S proteasome	O					[60]
PD	α-Synuclein A53T and A30P	Perturbation of CMA via blocakage of lysosomal translocation of substrates				O		[75]
HD	PolyQ-expanded huntingtin fragment	Autophagy dysfunction	O	O		O		[80, 81]
HD	PolyQ-expanded huntingtin fragment	Axonal transport dysfunction		O	O	O		[107– 109]
ALS	Mutant SOD1	Axonal transport dysfunction	O	O	O	O	Squid giant axon	[102–106]
IBMPFD/ALS	Mutant VCP	Protein degradation				O		[62]
**Mitochondria**								
AD	Amyloid beta	Amyloid beta binds to mitochondrial proteins such as ABAD and CypD to induce ROS generation, mPTP opening, and mouse behavioral defects	O	O		O		[112, 113]
AD	Amyloid precursor protein	Mitochondrial protein import dysfunction	O	O		O		[114, 115]
HD	PolyQ-expanded huntingtin	Defects in mitochondrial protein import, trafficking, MPTP opening, and calcium regulation	O	O		O		[122–125]
PD	Mutant and WT α-synuclein	VDAC blockage and mitochondrial protein import dysfunction	O			O	Rat, yeast	[129, 130]
ALS/FTD	Mutant and WT TDP-43	TDP-43 binds to respiratory complex I subunits and induce defects in mitochondrial protein translation	O	O		O	Yeast	[131, 132]
ALS/FTD	Poly-GR repeat protein	Poly-GR binds mitochondrial ribosomal proteins and induce defects in mitochondrial protein translation	O		O	O		[133]
**Stress granules**								
ALS	Mutant profilin 1	Altered SG dynamics				O	Yeast	[143]
ALS/MSP	Mutant hnRNPA1/A2	Altered SG dynamics	O		O	O		[144]
ALS/FTD	Mutant FUS	Altered SG assembly and dynamics				O		[145]
ALS/FTD	Mutant TIA1	Altered SG dynamics	O			O		[146]
ALS/FTD	Mutant and WT TDP-43	Altered SG dynamics				O		[147]
ALS	Mutant SOD1	Altered SG dynamics and morphology				O		[148]
IBMPFD/ALS	Mutant VCP	Altered SG quality control				O		[149]
HD	PolyQ-expanded huntingtin	Increased SG formation				O		[152]

The entry 'O' in Table 1 affirms the experimental models used to support the listed phenotypes for each diseases

## Protein toxicity in the nucleus

Nuclear inclusions (NIs) of toxic proteins in neurons are observed in approximately 20 different neurodegenerative diseases [13]. In particular, nuclear accumulation of toxic disease proteins is closely associated with the pathogenesis of polyQ diseases (see below for details). Although a growing body of evidence indicates nuclear dysfunction to be central to the pathogenesis of several neurodegenerative diseases, the precise role of neuronal intranuclear inclusion bodies in the disease pathogenesis is still a matter of debate. There is a view that microscopically visible NIs are not toxic, but are instead self-protective structures or incidental byproducts of the pathogenic process. This view proposes that the more soluble protofibrillar or oligomeric aggregates (as opposed to the more mature fibrillar aggregates formed inside the nucleus) have toxic properties in afflicted neurons [14–18]. Whether or not the nuclear inclusion bodies are the major source of nuclear protein toxicity, nuclear dysfunctions such as transcriptional alteration and impaired nucleocytoplasmic transport are evident in many cases of neurodegenerative diseases [19, 20].

As described above, polyQ diseases may be representative neurodegenerative diseases associated with nuclear protein toxicity [21]. There are at least nine polyQ diseases, including HD, dentatorubral–pallidoluysian atrophy (DRPLA), spinal bulbar muscular atrophy (SBMA), and the spinocerebellar ataxias (SCAs) 1, 2, 3, 6, 7, and 17 [7]. Each of these nine polyQ diseases is caused by CAG (Q) repeat expansion mutation in each of the disease-responsible genes [e.g., the *huntingtin* (*htt*) gene mutation for HD]. Upon expansion of the Q repeats, the disease-responsible proteins, most of which mis-localize to the nucleus, gain a propensity to aggregate and multimerize with numerous target proteins. For instance, although normally a cytoplasmic de-ubiquitinase protein, ataxin-3 predominantly localizes to the nucleus upon expansion mutation in SCA3. Similarly, various animal models present nuclear aggregation of mutant htt in neurons [22]. Of note, however, the nuclear accumulation of mutated polyQ proteins is not always closely associated with the pathogenesis of diseases. In case of SCA2, it has been shown that nuclear localization of the SCA2 protein is not necessary for SCA2 pathogenesis in mice or humans [23].

PolyQ NIs are often co-localized with ubiquitin, heat shock proteins, and numerous target proteins [21]. Some proteins, such as cAMP response element-binding protein (CREB)-binding protein (CBP), have been identified as the target of polyQ proteins in a number of different polyQ diseases, suggesting that their interaction with polyQ proteins may be dependent on the expanded Q repeat region, rather than the flanking regions, of the polyQ proteins. The detection of various transcription factors, such as CBP [24–27], TATA-binding protein (TBP) [28], nuclear co-repressor (NCoR) [29], and RE1-silencing transcription factor/neuron-restrictive silencer factor (REST/NRSF) [30], within polyQ NIs suggests a sequestration mechanism by which polyQ proteins may induce transcriptional dysregulation. Notably, many of the proteins sequestered by polyQ proteins function as epigenetic regulators that may be responsible for the system-wide transcriptional dysregulation in a subset of polyQ diseases [20, 31]. Consistently, a previous study reported that the mutant htt and ataxin-3 proteins could directly bind to histone acetyl-transferases, such as CBP and p300/CBP-associated factor (P/CAF), thereby impairing histone acetylation in neurons [32, 33]. However, the reduction of histone acetylation by mutant htt remains disputed [20]. Aside from histone modification, direct epigenetic changes to the DNA have also been reported in HD. Previous studies reported hypomethylation of DNA with CpG-poor regions in the HD cell culture model [34] and a decreased level of 7-methylguanine (7mG) by mutant htt proteins in mouse and human patient samples [35]. Furthermore, a direct interaction between mutant htt proteins and methyl-CpG-binding protein 2 (MeCP2) has been reported, the interaction of which enables mutant htt to bind directly to the methylated DNA regions [36].

Transcriptional and epigenetic alterations have been shown to contribute to the broad spectrum of neuronal phenotypes ranging from early neuropathic features to late-stage neuronal cell death in polyQ diseases [31]. For instance, recent studies showed that polyQ proteins induced early changes to the dendrite morphology through the perturbation of RNA granule formation and transcriptional cascades regulating the ER-to-Golgi (COPII) pathway [37–39]. In the SCA1 mouse model, the translational repressor Capicua was shown to be critically involved [40], and in HD and DRPLA mouse models, treatment with histone-deacetyltransferase (HDAC) inhibitors (sodium butyrate, 4-phenylbutyric acid sodium salt, and suberoylanilide hydroxamic acid) was shown to ameliorate neurotoxicity [41–44]. These results demonstrate a crucial contribution of transcriptional and epigenetic alterations in at least a subset of polyQ diseases.

In addition to polyQ diseases, transcriptional dysregulation is also observed in other neurodegenerative diseases, such as AD [45–47] and PD [48, 49], although they are not generally accompanied by nuclear accumulation of toxic proteins. Similar to polyQ diseases, AD and PD also manifest epigenetic alterations, though the mechanisms of which remain to be elucidated [20, 31]. Nevertheless, treatment with certain epigenetic drugs, such as HDAC inhibitors, ameliorated AD and PD phenotypes in animal models [31], indicative of the relevance and importance of epigenetic alterations in the disease pathogenesis.

In addition to the transcriptional and epigenetic alterations, nucleocytoplasmic transport defects have emerged as one of the principal nuclear dysfunctions manifested in neurodegenerative diseases such as ALS/FTD, HD, and AD [19, 50]. The mechanisms by which nucleocytoplasmic transport becomes disrupted range from sequestration of nuclear pore complex (NPC) molecules by toxic RNA or proteins [19, 51–56] to direct blockage of nuclear pores by toxic disease proteins [57]. Some excellent reviews on this topic have recently been published, which we recommend for detailed discussion [19, 50].

## Protein toxicity in the cytoplasm

Many of the disease proteins are prone to accumulate in the cytoplasm, in which the pool of potential target molecules differs significantly from that of the nucleus. For example, it is the cytoplasm in which the protein quality control (PQC) system mostly resides, not in the nucleus. The cytoplasm also contains a more elaborate cytoskeleton compared to the nucleus. Hence, due to the physical proximity, cytoplasmic protein toxicity can directly impinge on the PQC system and cargo transport via cytoskeleton disruption. In this section, we will focus on the cytoplasmic protein toxicity associated with the PQC system and the cytoskeleton.

The accumulation of misfolded proteins in neurodegenerative diseases inevitably burdens the PQC system, which comprises the ubiquitin–proteasome system (UPS), chaperone-mediated autophagy (CMA), macroautophagy, and ER-associated degradation (ERAD) [58]. UPS impairment is considered to be one of the major contributing factors of neurodegenerative disease pathogenesis. Previous studies showed that aggregated beta-sheet-rich prion proteins and aggregated Tau in AD could block the 20S and 19S proteasome particles, respectively, which impaired UPS-mediated degradation [59, 60]. Consistently, genetic mutations of UPS components such as E3 ligase Parkin, deubiquitinating enzyme ubiquitin carboxy-terminal hydrolase L1 (UCH-L1), and ATPase valosin-containing protein (VCP), can lead to neurodegeneration [61, 62]. In addition, overexpression of certain components of UPS could ameliorate the disease phenotypes in neurons in many neurodegenerative disease models [63–66]. For example, PD-associated G2019S LRRK2 mutant proteins can be ubiquitinated by E3 ligase C-terminus of HSP70-interacting protein (CHIP), whose overexpression enhances the ubiquitin proteasomal degradation of LRRK2 mutant proteins [67]. Consistently, CHIP knockout mice displayed exacerbated polyQ pathology [68]. Furthermore, mutant htt has been shown to undergo ubiquitin proteasomal degradation via E3 ligase UBE3A [69], the activity of which is down-regulated by UBR5 [70], a genetic modifier for HD [71]. Moreover, most of the protein inclusions in neurodegenerative diseases are positive for ubiquitin and chaperones, both of which become depleted in the afflicted neurons [72]. Conversely, a recent study by Nucifora and colleagues suggested that ubiquitination could be a mechanism by which protein inclusions are formed [73]. They showed that WSB1 could induce aggregation of G2019S LRRK2 via K27 and K29 ubiquitination, which appeared to be neuroprotective [73]. Ubiquitination may thus protect against protein toxicity by either inducing degradation or aggregation of toxic proteins.

CMA is a selective protein degradation system that eliminates proteins harboring a pentapeptide KFERQ-like motif, which is found in approximately 30% of cytosolic proteins [58]. When folded properly, the KFERQ motif is not exposed to the surface. However, misfolding of these proteins exposes the motif that can be subsequently recognized by the heat shock cognate protein 70 (HSC70) chaperone and CMA adaptor lysosomal membrane-associated protein 2A (LAMP-2A). Several disease-associated proteins such as LRRK2 and α-synuclein also harbor KFERQ-like motifs that are recognized by CMA for degradation [74, 75]. A previous study showed that α-synuclein proteins in PD could bind to LAMP-2A with an unusually high affinity. This strong binding in turn resulted in a “traffic jam” during cargo translocation across the lysosomal membrane, thereby inhibiting CMA [75]. As for LRRK2, its binding to the lysosomal membrane is enhanced by certain mutations, thereby facilitating accumulation of α-synuclein among other CMA substrates [74]. Moreover, PD-associated mutations in UCHL1 also interfere with the CMA process [76]. These results suggest that CMA is one of the central processes by which PD-associated proteins are degraded and that interfering with the CMA process may result in α-synuclein accumulation. In a few other studies, the augmentation of CMA was shown to enhance the removal of pathogenic disease proteins in various neurodegenerative diseases [77–79], suggesting that CMA may be an important therapeutic target for diseases associated with protein toxicity. Since aggregation-prone proteins can be efficiently eliminated by macroautophagy, its role in neurodegenerative diseases has been extensively pursued. In HD, macroautophagy activity is reduced due to the failure in cargo recognition by autophagic vacuoles [80]. In addition, a certain species of mutant htt proteins has been shown to be selective-autophagy resistant, likely due to its unconventional conformation that is unfavorable for cargo recognition by autophagic vacuoles [81]. In many neurodegenerative diseases, autophagy can be induced as a compensatory response to the failure of UPS in afflicted neurons [82–84]. However, it appears that the compensatory induction of autophagy is not enough to overcome the accumulation of ubiquitin-positive toxic proteins in HD. Consistent with this, it was shown that further genetic or pharmacological activation of autophagy has obvious therapeutic benefits in various disease models [85].

Protein toxicity commonly produces ER stress; in turn, ER stress can cause up-regulation of chaperones, ERAD and apoptotic genes, global protein translational arrest, and stress granule formation [86]. ER stress can be caused in a number of ways; one of these causes is ERAD failure. For instance, VCP, a necessary component of ERAD, was shown to be sequestered by mutant htt [87, 88]. In another study, overexpression of VCP was shown to rescue ERAD failure caused by mutant htt [89]. Interestingly, the sequestration of VCP by polyQ proteins occurs in at least four other polyQ diseases (SCA1, SCA3, SCA7, and SBMA) [90, 91], in which the loss of VCP function may be a common pathogenic mechanism.

Pathological inclusions of cytoskeletal proteins, such as neuronal intermediate filament (IF) proteins or the microtubule-associated protein tau (MAPT), are neuropathological signatures in various neurodegenerative diseases [92]. Specifically, tau-associated microtubule defects are linked to a range of neurodegenerative diseases known as “tauopathies” [93]. Changes in F-actin structures have also been reported in polyQ diseases [38] and AD [94]. Furthermore, formation of ADF/cofilin-actin filament bundles (rods) that can occlude neurites and block vesicle transport has been implicated in neurodegenerative diseases [95]. In addition to these changes in cytoskeletal structures, accumulation of toxic disease proteins can lead to defects in axonal transport [96–101]. For example, defective axonal transport was reported to be a key early feature of pathogenesis prior to neurodegeneration in various SOD1 animal models of ALS [102–106]. Various animal models of HD also showed abnormalities in both anterograde and retrograde axonal transport [107–109].

Cytoplasmic protein toxicity encompasses a whole array of neuronal phenotypes, many of which are shared among neurodegenerative diseases. Hence, therapeutically neutralizing cytoplasmic protein toxicity may be beneficial, provided that the toxic proteins remain static in the cytoplasm. However, from the cytoplasm in which toxic disease proteins are first made, these proteins can be transported to other organelles such as the nucleus (discussed above), the stress granules (discussed later), or the mitochondria (discussed next), all of which can complicate any attempts to remedy cytoplasmic protein toxicity. Thus, closer examination of protein toxicity in the organelles in which toxic proteins tend to accumulate is warranted.

## Protein toxicity in the mitochondria

The importance of the mitochondria to cell survival can easily be envisaged, as they are the organelles primarily responsible for ATP production in eukaryotic cells. Thus, mitochondrial dysfunction can be detrimental for cell survival, which can be catastrophic particularly to the brain, for the following reasons. First, most neurons cannot be replaced and thus need to be maintained due to their post-mitotic nature. This will inevitably lead to the accumulation of mitochondrial toxicity, by which the irreplaceable neurons will eventually succumb to death. Second, the excitability of neurons allows for significant influx of calcium ions that are buffered by mitochondria, the dysfunction of which will lead to excitotoxicity. Third, the elongated morphology of neurites entails a local supply of ATP by the mitochondria, the dysfunction of which will perturb growth and maintenance of neurites [110]. Hence, it is not surprising that mitochondrial dysfunction is one of the cardinal features of neurodegenerative diseases.

Mitochondrial dysfunction can be both primary and secondary drivers of neurodegeneration. In this section, we will mainly deal with the cases in which mitochondrial dysfunction is clearly a direct primary consequence of protein toxicity in the mitochondria. The following six toxic disease proteins that accumulate in mitochondria will be discussed: amyloid beta, amyloid precursor protein (APP), α-synuclein, mutant htt, TDP-43, and poly-GR DPRs.

Extracellular amyloid beta accumulation is one of the key pathological hallmarks of AD, in which mitochondrial dysfunction is often observed [111]. No direct mechanistic link between amyloid beta and mitochondrial dysfunction was identified until Lustbader et al. showed in 2004 that amyloid beta can localize to the mitochondria and directly bind to amyloid beta-binding alcohol dehydrogenase (ABAD) to induce mitochondrial toxicity [112]. Amyloid beta has also been shown to interact with cyclophilin D (CypD), an integral component of the mitochondrial permeability transition pore (mPTP), which sensitizes the opening of mPTP in both AD patients and mAPP mice brains [113].

APP, from which amyloid beta is derived, has also been shown to produce mitochondrial toxicity in models of, and patients with, AD. Anandatheerthavarada and colleagues showed that APP has a leader sequence with which APP localizes to the mitochondria in HCN-1A cells. The large acidic domain residues of APP (220–290) were found to clog the pores of TOM40 and TIM23, mitochondrial translocase of outer and inner membrane, respectively [114, 115]. When the authors experimented with postmortem human brain samples, they found that mitochondrial APP was observed only in AD brains [115]. Why APP does not localize to mitochondria under normal condition is currently unknown. In any case, this evidence strongly suggests that physical interaction of APP and amyloid beta with mitochondrial proteins is sufficient to generate oxidative stress, reduce ATP production, depolarize mitochondrial membrane potential, and sensitize mPTP opening, all of which contribute strongly to the mitochondrial dysfunction manifested in AD [110, 111, 116]. A recent study that shows reduction in amyloid beta toxicity by promoting mitochondrial proteostasis underscores the contribution of mitochondrial dysfunction in AD pathogenesis [117].

Mitochondrial dysfunction is not unique to AD. In HD, an energy-deficit related to mitochondrial dysfunction was first observed more than two decades ago [118]. The mechanisms by which mutant htt proteins induce mitochondrial dysfunction have been shown to be as diverse as that in AD. Aside from the mutant htt perturbing transcription of genes related to mitochondrial biogenesis and function in the nucleus [119, 120], it could also directly interact with mitochondrial proteins [121]. The N-terminal fragment of mutant htt localizes to the mitochondria [122–124] both in vivo and in vitro, and it interacts with the TIM23 complex, thereby clogging the mitochondrial import process [125]. These toxic interactions of mutant htt with mitochondrial proteins perturb calcium regulation, sensitize mPTP opening, depolarize mitochondrial membrane potential, and ultimately lead to neuronal demise [122–125].

Many genetic mutations linked to PD have been shown to cause mitochondrial dysfunction [126]. α-Synuclein, which is the central aggregating component of the Lewy bodies found in PD and Lewy body diseases, has high affinity for negatively charged lipids, including mitochondrial membranes [127–129]. In addition, α-synuclein has been shown to bind to several mitochondrial proteins such as the voltage-dependent anion channel (VDAC) in a monomeric form [129] and to TOM20 in an oligomeric form [130]. These interactions hinder the exchange of ATP/ADP between the mitochondria and the cytosol and impair mitochondrial protein import, both of which undermine mitochondrial function [129, 130].

ALS and FTD are diseases that manifest different clinical symptoms and yet share overlapping etiology. The pathological hallmark of ALS/FTD is the cytoplasmic mis-localization of TDP-43, but the mechanism by which TDP-43 proteins cause toxicity in the cytoplasm remains unclear. Wang et al. proposed a novel mode of toxicity by showing that TDP-43 possesses internal mitochondrial targeting signals that can direct TDP-43 into the mitochondria. The mitochondrial targeting becomes enhanced in ALS or FTD patients, which perturbs oxidative phosphorylation by means of binding to mitochondria-transcribed ND3 and ND6 mRNA and prohibiting their translation [131]. Conversely, Kawamata et al. reported that disease-associated mutant TDP-43 (TDP43 A315T) expression did not lead to any aberrant mitochondrial functions aside from calcium dysregulation [132]. These conflicting data warrant further investigation for us to assess more accurately the potential relevance of the mechanism described above. Interestingly, one of the arginine-rich DPRs (poly-GR repeats) derived from the hexanucleotide expansion mutation of *C9ORF72* has also been shown to localize to the mitochondria and interact with mitochondrial ribosomal proteins, thereby causing mitochondrial dysfunction [133]. These recent findings suggest the mitochondria to be the primary driver of neurodegeneration in ALS/FTD as well.

How and for what purpose do these disease-associated toxic proteins accumulate in the mitochondria? Such heterogeneity of disease-associated proteins targeting mitochondria suggests non-specific mechanisms in which mitochondria act as cellular waste bins for toxic and presumably misfolded disease proteins. Ruan et al. recently proposed, in a rather timely manner, the mechanism by which misfolded cytoplasmic proteins accumulate inside the mitochondria to be degraded [134]. Ruan et al. showed that upon heat stress, misfolded cytoplasmic proteins enter mitochondria via mitochondrial translocase Tom70/Tom40 and are degraded by Pim1 in yeast [134]. Given that most toxic disease proteins are prone to misfolding, the potential relevance of this mechanism may be far-reaching in understanding the mitochondrial pathology common to most neurodegenerative diseases.

## Protein toxicity in the stress granules

Neurons undergoing degeneration display immense stress to which multifaceted responses are launched to mitigate it. One of the key processes that occur in response to cellular stress is the formation of stress granules (SGs) [135]. Upon stress induction, cap-dependent translational processes are aborted and the messenger ribonucleoproteins (mRNPs) disengaged from the ribosomes begin to coalesce [136]. The RNA-binding proteins (RBPs) in these mRNPs interact electrostatically with one another through low complexity domains (LCDs) [137]. These interactions eventually facilitate liquid–liquid phase separation (LLPS) from the cytoplasm, thereby forming SGs. Concomitant to the formation of SGs, chaperones such as HSP70 are up-regulated via m^6^A-mediated cap-independent translation [138] to defuse stress by promoting refolding or degradation of misfolded proteins. Once the stress is resolved, the chaperones [139], along with autophagy [140], become instrumental in the disassembly of SGs [141].

In some neurodegenerative diseases, such as ALS or FTD, the SGs are infiltrated by disease-associated proteins that elicit an improper stress response. Many of the ALS genes encode proteins that are associated with SGs, such as Profilin-1, hnRNPA1/A2, fused in sarcoma (FUS), T cell-restricted intracellular antigen-1 (TIA1), and TDP-43, several of which are also linked to FTD [135, 142–147]. Increased cytoplasmic concentration or a mutation in the LCD of these proteins seems conducive to the initial LLPS, with subsequent stabilizing effect of SGs beyond the physiological necessity. The stabilized SGs that persist may then evolve into pathological fibrils [141].

Other ALS or FTD-associated proteins, such as superoxide dismutase-1 (SOD1) and valosin-containing protein (VCP), also impinge on the SGs, albeit by mechanisms that are independent of LCD [136, 148]. SOD1 variants associated with ALS form aggregates around the SGs, which suggests that SG formation precedes SOD1 aggregation [148]. Encapsulated by mutant SOD1, SGs display reduced dynamics and irregular morphology [148]. Such perturbed SG dynamics can be effectively combatted by the PQC system, of which VCP is a prominent member [136]. VCP is an ubiquitin segregase that uses ATP to extract ubiquitinated proteins from complexes to which they belong [136]. Stress induces SUMOylation of the VCP N-terminal domain by Ubc9, and it is one of the mechanisms by which VCP localizes to the SGs [149]. Mutations of VCP in the N-terminal domain thus have been shown to hinder SUMOylation, the modification without which hampers VCP from infiltrating SGs to extract ubiquitinated misfolded proteins for degradation [149]. Hence, SG disassembly fails with VCP mutation and the property of these SGs slowly transforms from dynamic liquid droplet like to pathological fibrils.

Whether or not SG pathology is associated with HD is still controversial. One study showed that mutant htt forms stress bodies, but not SGs [150]. Another study showed that both normal and mutant htt proteins can bind to SG-related factors such as Caprin-1 and G3BP1 [151]. Under normal conditions, neither normal nor mutant htt co-localized with the SG marker TIA1, whereas after arsenite treatment, both normal and mutant htt co-localized with TIA1; no difference in SG dynamics could be observed between normal and mutant htt in striatal precursor cells with or without arsenite treatment [151]. Similarly, another study reported that both normal and mutant htt interact with Caprin-1 and G3BP1; however, this study showed that the size and number of SGs were larger in striatal precursor cells expressing mutant htt compared to cells expressing normal htt [152]. These studies focused on mis-localization of mutant htt into SG to disrupt its dynamics; the results of these studies were mixed. Some studies suggest that rather than mutant htt localizing to SGs to cause their dysfunction, SG-related factors may mis-localize to mutant htt inclusions [153]. Time-lapse images in AD293 cells showed the formation of mutant htt inclusions with subsequent TIA-1 recruitment [154]. Another study showed that less than 1% of the interactors of mutant htt also interacted with SGs [155], which seems to support the view that mutant htt inclusions recruit certain SG-related factors.

Although the link between SG pathology and neurodegeneration has been well established, there is a lack of literature on the mechanism by which pathological SGs precipitate neurodegeneration. It has been a commonly accepted notion that SG formation upon stress induction contributes substantially to the global translational shutdown. However, a recent study using RNA-sequencing and single-molecule fluorescence in situ hybridization (smFISH) showed that only 10–12% of total mRNA molecules are localized to SGs [156], which does not support the notion that SGs are indispensable for global suppression of translation. Indeed, a previous study showed global translational shutdown upon stress induction in cells with G3BP mutations that prohibit SG formation [157]. This evidence supports the notion that SGs are dispensable for global suppression of translation. If it is not global translational shutdown, what then is the major function of SGs during stress, and how do pathological SGs precipitate neurodegeneration? Several studies showed that SG formation could impinge on intracellular signaling by sequestering key signaling molecules such as mammalian target of rapamycin (mTORC1) [135, 158, 159]. Thus, one of the mechanisms by which pathological SGs precipitate neurodegeneration may be through chronic impediment of intracellular signaling. Henceforth, elucidating the mechanistic link between pathological SGs and neurodegeneration should be one of the major focal points of research.

## Propagation of toxic disease proteins

**Fig. 2:**
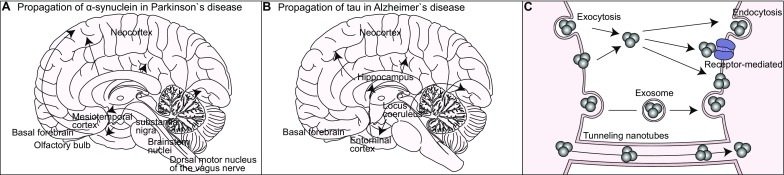
Propagation of misfolded proteins via intercellular transmission. Gradual change in the distribution of α-synuclein (**a**) and tau (**b**) inclusions in the brains of patients suffering from Parkinson’s (**a**) and Alzheimer’s (**b**) diseases. **c** Intercellular transmission of misfolded proteins via exocytosis, endocytosis, exosomes, and tunneling nanotubes

One of the interesting features often observed in neurodegenerative diseases is the gradual expansion of brain regions affected by pathogenic protein aggregates over time. In postmortem brains of PD patients, histopathological analyses have revealed the stereotypical progression of pathogenic inclusions from the autonomic nervous system, and from the dorsal motor and anterior olfactory nuclei to the substantia nigra, basal forebrain and the locus coeruleus, as well as to the hippocampus, neocortex, and basal ganglia [160] (Fig. 2a). In the postmortem brains of AD patients, tau inclusions initially appear in the transentorhinal cortex and later emerge in the hippocampal formation and neocortex [161] (Fig. 2b). These observations have led to an intriguing hypothesis that the expansion of the damaged brain regions is due to the gradual “prion-like” intercellular transmission of aggregates rather than the cell-autonomous accumulation of neuronal aggregates [162–164]. Supporting this hypothesis, clinical studies have shown that healthy embryonic mesencephalic neurons implanted into the striatum of patients with advanced PD developed scattered α-synuclein- and ubiquitin-positive inclusions many years after transplantation [165]. Similarly, healthy neurons implanted into the striatum of transgenic mice overexpressing human α-synuclein exhibited an accumulation of Lewy body-like inclusions [166–169]. In addition, the implementation of either patient-derived fibroblasts or pluripotent stem cells carrying mutant htt into the brain of neonatal wild-type mice was shown to induce cell-to-cell propagation of the mutant protein, a progressive loss of host cells, and behavioral deficits characteristic to HD [170]. These results suggest that pathological aggregates can transfer between diseased and healthy cells in humans and animals.

The more direct evidence supporting the mobility of aggregates between cells has been provided by studies either employing an intracerebral application of exogenous aggregates derived from diseased humans and animals or ectopic overexpression of α-synuclein and tau in a population of neurons to examine whether the aggregates can spread through the brain connectome [166, 171–173]. For example, an intracerebral injection of brain extracts prepared from the symptomatic P301S tau transgenic mice was shown to be sufficient for inducing neurofibrillary tangles in presymptomatic P301S tau transgenic mice at the injection site as well as in the distant brain regions that are physically separated by one or more synapses from the injection site [166, 174]. Similarly, within 6–12 months after the inoculation of amyloid beta-containing brain extracts derived from either AD patients or aged APP transgenic mice into the hippocampus and neocortex of young APP transgenic mice, amyloid beta deposition and its associated pathology were widespread in the brain [175–177]. Likewise, an intracerebral administration of brain or spinal cord homogenates prepared from symptomatic α-synuclein transgenic mice facilitated the appearance and spread of Lewy pathology in presymptomatic recipient transgenic mice [171]. The spread of pathological changes was recapitulated by a local injection of synthetic α-synuclein fibrils or tau filaments in presymptomatic transgenic mice, suggesting that aggregates, but not other factors in the brain homogenates, are sufficient for the spreading of the pathological changes in the brain [171, 178–180]. Finally, selective overexpression of transgenic tau, amyloid beta, or α-synuclein in a population of neurons could trigger the spread of misfolded proteins to the interconnected brain regions in transgenic mice [181–186].

Several lines of evidence suggest that peripherally introduced aggregates can lead to the accumulation of misfolded proteins in the central nervous system (CNS). For instance, transgenic mice expressing a mutant human α-synuclein exhibited pathogenic inclusions and neuroinflammatory responses throughout the CNS within 2–4 months after an intramuscular injection of recombinant α-synuclein fibrils. Those animals also displayed a debilitating motor impairment, which is one of the clinical symptoms characteristic of PD [187]. However, when the sciatic nerve, which connects the muscles to the spinal cord, is severed in those same mice, the development of the pathogenic inclusions and neuroinflammatory responses in the CNS was significantly delayed. This suggests that the retrograde transport of misfolded proteins via the peripheral nerve is required for disease propagation, at least in this mouse model. A recent study has shown that α-synuclein fibrils injected into the olfactory bulb of wild-type mice propagate transneuronally to distant brain regions and induce progressive olfactory deficits [188]. Similarly, mutant htt ectopically expressed in sensory receptor neurons in *Drosophila* can spread transcellularly to neuronal and glial cells in the brain [189–191]. Another study has demonstrated that an intestinal application of either the brain lysate from human PD patients or recombinant α-synuclein in rats could elicit α-synuclein inclusions in the dorsal motor nucleus of the vagus nerve in the brainstem [192]. Moreover, systemic treatment of aggregates, such as repeated injections of α-synuclein fibrils into the tail vein and an intraperitoneal inoculation of tau extracts or amyloid beta seeds, was sufficient to cause accumulation of deposits in the brain [193–196].

There are several varying molecular mechanisms by which pathogenic aggregates can transfer between cells (Fig. 2c). Exocytosis is one of the main secretory mechanisms involved in releasing aggregates from donor cells, which occurs in an intracellular calcium- and endosome-dependent manner [197–199]. Alternatively, the misfolded proteins can be released into the extracellular space within secretory vesicles called exosomes [200–202]. Exosomes are vesicles of 50–100 nm diameter that normally mediate intercellular transportation of mRNA, small regulatory RNA, and specific proteins between the cells [203]. A number of studies have demonstrated that exosome-mediated propagation is implicated in the spreading of pathogenic inclusions in neurodegenerative diseases. Studies using immunofluorescence and immunoelectron microscopy have revealed that the exosomes are associated with amyloid beta peptides, phosphorylated tau, and other related molecules [204]. Furthermore, exosomes isolated from diseased transgenic animals or human patients were shown to have an ability to nucleate oligomerization of endogenous proteins in recipient cells [205, 206]. Accordingly, pharmacological inhibition of key regulatory enzymes mediating secretion and synthesis of the exosomes significantly reduced both the amyloid plaque formation in the AD mouse model and the secretion and propagation of tau from microglia in vitro and in vivo [182, 207]. Finally, pathogenic inclusions can be transferred through tunnel-like structures called tunneling nanotubes that connect the cytosolic compartments between neighboring cells to facilitate intercellular material exchange for communication [208]. The diameter of nanotubes ranges from 50 to 200 nm, and their lengths can reach up to several cell diameters [209]. In vitro studies have shown that α-synuclein fibrils can be transferred via tunneling nanotubes to lysosomes of recipient cells, such as mouse catecholaminergic cells and human primary brain pericytes, and subsequently induce the aggregation of cytosolic α-synuclein [208, 210].

In sum, the progressive accumulation of specific protein aggregates along anatomical connections is a common hallmark of major neurodegenerative diseases such as AD and PD. Extensive evidence from in vitro and in vivo studies suggests that one of the fundamental pathogenic mechanisms by which neurodegeneration transpires is the intercellular transmission of protein aggregates in synaptically connected brain networks.

As describe above, disease propagation model is supported by a number of preclinical evidence, but there are also some observations that cannot be fully explained by this model. For example, a fetal graft implanted in some PD patients was found to be without pathology in autopsies performed two decades following transplantation [211, 212]. In addition, proteins associated with neurodegenerative diseases are unlikely to transmit between individuals as a disease-causing infectious agent [213, 214]. Finally, a recent study showed that brain regions manifesting Lewy pathology neither fully correlate with the synaptic connection patterns revealed by connectome mapping [215] nor follow the spatiotemporal spread patterns described by Braak et al. Thus, further research is required to fully understand the clinical relevance of the aggregate propagation model versus the cell-autonomous model.

## Selective neuronal vulnerability

Most of the genes whose mutations cause neurodegenerative diseases are ubiquitously expressed in all developmental stages of life. However, developmental defects are minimal in patients with neurodegenerative diseases such as AD, HD, PD, or ALS. In addition, neurodegenerative disease patients tend to manifest late-onset, cell-type-specific neurodegeneration [216]. Due to their post-mitotic nature, neurons may be more vulnerable to cellular toxicity than other cell types which are capable of regeneration. Furthermore, neurons are generally more ATP dependent than other cell types, rendering neurons more vulnerable to energy crises caused by membrane potential changes and mitochondrial dysfunction. Nevertheless, two important questions remain to be answered. First, what accounts for the selective neuronal toxicity? Second, why does such toxicity stay dormant during development, but become damaging with age?

Both sporadic and familial disease cases present with selective neuronal vulnerability. This selective neuronal vulnerability is often indistinguishable between patients with sporadic and familial etiology, but is distinct from disease to disease [217]. Hence, we speculate that selective neuronal vulnerability may arise from genetic predisposition or environmental factors that chiefly affect certain neurons. For instance, PD is often associated with mutations in genes that are involved in mitochondrial function and also with exposure to environmental mitochondrial toxins [126]. Whether caused by genetic or environmental factors, PD involves selective degeneration of the substantia nigra pars compacta (SNpc). Thus, we deduce that the SNpc may be particularly vulnerable to mitochondrial dysfunction. What makes SNpc especially vulnerable to mitochondrial dysfunction is unclear, though the unique properties of those neurons, such as the oxidation of dopamine neurotransmitters and the pacemaking activity of Ca_v_1.3 L-type Ca^2+^ channels [218], are likely contributors. Nonetheless, the possibility of other factors contributing to the selective SNpc degeneration should not be excluded.

Similarly, both sporadic and familial ALS are associated with RNA metabolism [219], the dysfunction of which may selectively render upper and lower motor neurons vulnerable to degeneration. Interestingly, RNA metabolism is also compromised by the activation of human endogenous retrovirus k [220], which is associated with ALS [221]. Hence, RNA metabolism dysfunction may be associated with ALS, but whether it can cause selective motor neuron degeneration is still unclear.

PolyQ disease patients tend to exhibit cerebellar atrophy [222]. This outcome suggests that the cerebellum is particularly vulnerable to protein toxicity mediated by the expanded polyQ proteins. We speculate that the cerebellum may have a weaker defense system against polyQ toxicity or that it expresses a disproportionate amount of proteins that are polyQ targets. Interestingly, fetal alcohol exposure primarily causes cerebellar pathology, which is linked to reduced CBP expression in the cerebellum [223]. In addition, Rubinstein–Taybi syndrome, which is caused by a CBP loss-of-function mutation, involves cerebellar pathology [224]. Since many different polyQ proteins have been shown to sequester and to interfere with CBP [225, 226], we speculate that a polyQ-induced loss of CBP function may contribute to the selective cerebellar pathology in polyQ diseases.

There are many possible explanations for minimal developmental defects in patients who later develop neurodegenerative diseases. We believe that the following are the three most viable explanations: (1) the PQC system may mitigate protein toxicity early in life but may fail later in life, (2) protein toxicity eventually reaches a critical threshold, beyond which defense mechanisms start to collapse, or (3) environmental factors or epigenetic alterations during and after development contribute to the disease onset later in life. We believe that all of these processes may contribute to the late onset of neurodegenerative diseases. Thus, we propose that development is the critical window within which therapeutics should be applied to prevent or delay disease initiation.

## Discussion and future perspectives

Neurodegenerative diseases, for which there are no remedies, correlate well with age, and this is a major conundrum with which we are confronted in an aging society. With years of massive research efforts carried out in laboratories around the globe, much knowledge of the nature of neurodegenerative diseases has been accrued with only a minimal progress in the actual development of effective therapeutics. To bridge the gap between our current understanding of the disease and the application thereof to the development of effective therapeutics, in this review, we have systematically analyzed and summarized the mechanistic underpinnings of protein toxicity (Table 1; Fig. 1), which is central to the development and progression of a vast array of neurodegenerative diseases such as AD, PD, ALS, FTD, and HD. We have discussed a number of toxic disease proteins within their respective subcellular contexts in an attempt to compare and contrast their pathogenic mechanisms in a localized area.

In this review, we have focused on the mechanisms of protein toxicity in neurodegenerative diseases, but protein toxicity can also be observed in psychiatric disorders such as schizophrenia. Recently, schizophrenia has been associated with genes such as *Neuronal PAS Domain Protein 3* (*NPAS3*), *Disrupted*-*in*-*schizophrenia 1*(*DISC1*), and *TRIO binding protein*-*1* (*TRIOBP*-*1*); translocation or point mutations in these genes may cause protein aggregation [227–229]. NPAS3-V304I proteins form aggregates, into which normal NPAS3 proteins are sequestered; NPAS3 loss of function then leads to decreased transcription of its downstream target, VGF [228]. A *DISC1* translocation mutation produces a truncated DISC1 protein, which can form aggregates and can act in a dominant negative manner. Three polymorphisms of *DISC1* have also been associated with major depression and schizophrenia [229]. TRIOBP-1 has been found in insoluble aggregates within brain lysates of schizophrenia patients’ brains. Amino acids 324–348 of TRIOBP-1 are thought to be critical for aggregation; TRIOBP-1 aggregation may affect actin dynamics and neurite growth [230]. Interestingly, the *TRIOBP* mutation is associated with deafness, which is often associated with psychiatric disorders. One study identified a family with schizophrenia and hearing impairment; for this family, the locus in which the causative mutation lies includes *TRIOBP* [231]. In addition to NPAS3, DISC1, and TRIOBP-1, CRMP1 and dysbindin can also form protein aggregates in schizophrenic patients [229, 232]; therefore, we infer that protein toxicity may be one of the mechanisms by which schizophrenia occurs. Interestingly, schizophrenia has been suggested to be linked to polyQ diseases as well [233]. It has been shown biochemically that DISC1 binds to mutant htt more strongly than it binds to normal htt [234]. This binding sequestered DISC1 away from PDE4, thereby increasing its activity. Overexpressing modified DISC1, which can interact with PDE4 but not with mutant htt, ameliorated anhedonia in a mouse model of HD [234]; anhedonia is one of the core features of schizophrenia. Many neurodegenerative disease patients also display mental or psychiatric symptoms such as depression and hallucinations [235, 236]; however, the molecular link between neurodegeneration and psychiatric symptoms remain undefined.

Increased life expectancy and the prevalence of neurodegenerative diseases in the twenty-first century are driving therapeutic research. However, currently there are only palliative drugs available to treat these diseases. The task of drug development is formidable; it has been estimated that AD drug development efforts face a 99.6% failure rate [237]. Hence, Pfizer (one of the leading pharmaceutical companies) recently announced its exit from the field of neuroscience [238]. However, as basic research is slowly helping us to understand the complexity of the brain, new treatment strategies against neurodegenerative diseases are being formulated.

One of the fastest-growing treatment strategies is antibody utilization [239]. For neurodegenerative diseases that involve protein toxicity, elimination of toxic proteins is an efficient way in which toxicity can be mitigated. Hence, antibodies against toxic disease proteins such as α-synuclein and amyloid beta are being developed. Recently, Biogen Inc. developed aducanumab, which was shown to reduce both amyloid plaques and cognitive decline in patients with mild form of AD after a 12-month trial [240]. However, another amyloid beta antibody (solanezumab) did not mitigate cognitive decline or reduce amyloid plaque in AD patients [241]. There are a few explanations that may account for this discrepancy. First, solanezumab administration may have been below the effective dose. Second, solanezumab may have bound to the wrong target. Third, the disease of patients in the solanezumab study may have been too advanced for the treatment to have been beneficial. Although only approximately 0.1% of the antibodies are known to traverse the blood–brain barrier [239], intravenous infusion of 400 mg every 4 weeks [241] seems to be a substantial dosage. Solanezumab targets amyloid beta monomers [241], whereas aducanumab targets oligomers and fibrils [240]; recent studies suggest that the oligomeric form may be the most toxic form [242]. This suggests that therapeutic target may have been at fault. Nevertheless, disease progression could perhaps be delayed if solanezumab was administered before any substantial oligomers or fibrils were formed. In any case, we can learn from these two examples, which highlight the significance of identifying the key drug target, correct dosage, and the disease stage at which to intervene.

Antibody treatment has its own drawbacks, however. As they cannot freely traverse across membranes, intracellular targeting of antibodies is very difficult, and intra-organellar targeting, even more so. Thus, antibody-based treatments have been more successful with extracellular targets (such as amyloid plaques) instead of intracellular targets (such as mutant htt and α-synuclein). Nevertheless, there are a few notable antibody-based drugs (RO7046015 from Roche and BIIB054 from Biogen) undergoing clinical tests targeting cell-to-cell transmission of α-synuclein [243]. Although there are various methods whereby antibody-based drugs can be delivered intracellularly in vitro and ex vivo [239], delivery in vivo often still poses insurmountable challenges. Hence, we believe that undertaking the challenge of target-specific delivery will be crucial in advancing the development of effective therapeutics against neurodegenerative diseases.

Our review discussed the mechanisms and the sites at which protein toxicity occurs to assist in the identification of druggable targets. We have also briefly discussed potential mechanisms of cell-to-cell propagation of toxic proteins and selective neuronal vulnerability in neurodegenerative diseases. We hope that by enhancing our understanding in these areas of research, more effective therapeutic strategies will be developed in the future.
